# Psychosocial Syndemics and Multimorbidity in Patients with Heart Failure ^†^

**DOI:** 10.20900/jpbs.20210006

**Published:** 2021-04-13

**Authors:** Kenneth E. Freedland, Judith A. Skala, Robert M. Carney, Brian C. Steinmeyer, Michael W. Rich

**Affiliations:** 1 Department of Psychiatry, Washington University School of Medicine, 4320 Forest Park Avenue, Suite 301, St. Louis, MO 63108, USA; 2 Department of Medicine, Washington University School of Medicine, 660 S. Euclid Avenue, St. Louis, MO 63110, USA

**Keywords:** comorbidity, health status disparities, heart failure, mental disorders, multimorbidity, patient readmission, self-care, self-management, social determinants of health, syndemic

## Abstract

Heart failure (HF) is a common cause of hospitalization and mortality in older adults. HF is almost always embedded within a larger pattern of multimorbidity, yet many studies exclude patients with complex psychiatric and medical comorbidities or cognitive impairment. This has left significant gaps in research on the problems and treatment of patients with HF. In addition, HF is only one of multiple challenges facing patients with multimorbidity, stressful socioeconomic circumstances, and psychosocial problems. The purpose of this study is to identify combinations of comorbidities and health disparities that may affect HF outcomes and require different mixtures of medical, psychological, and social services to address. The syndemics framework has yielded important insights into other disorders such as HIV/AIDS, but it has not been applied to the complex psychosocial problems of patients with HF. The *multimorbidity* framework is an alternative approach for investigating the effects of multiple comorbidities on health outcomes. The specific aims are: (1) to determine the coprevalence of psychiatric and medical comorbidities in patients with HF (*n* = 535); (2) to determine whether coprevalent comorbidities have synergistic effects on readmissions, mortality, self-care, and global health; (3) to identify vulnerable subpopulations of patients with HF who have high coprevalences of syndemic comorbidities; (4) to determine the extent to which syndemic comorbidities explain adverse HF outcomes in vulnerable subgroups of patients with HF; and (5) to determine the effects of multimorbidity on readmissions, mortality, self-care, and global health.

## INTRODUCTION

More than six million Americans have heart failure (HF), and the prevalence is expected to increase 46% by 2030. HF is one of the most common causes of hospitalization, impairment, and mortality in older adults. The annual cost of HF care is expected to increase 127% by 2030, to almost $70 billion in current dollars [1].

One of the reasons why HF is so burdensome and costly is that it is usually complicated by multimorbidity [2], especially in older patients [3]. Both cardiovascular and noncardiovascular disorders that are risk factors for HF tend to persist after HF has developed, and additional comorbidities tend to accumulate as patients age. Unfortunately, most HF treatment trials, as well as many other studies of HF, have excluded patients with complex medical and psychiatric comorbidities, physical or cognitive impairments, or frailty. This has left large gaps in research on the care of older adults with HF [4].

Many epidemiological and clinical studies have focused on common comorbidities in HF. Some have been broad investigations of multiple disorders (e.g., [5–7]), but most have focused on specific illnesses. This applies both to medical comorbidities such as diabetes [8] and psychiatric comorbidities such as depression [9]. In addition, research on socioeconomic factors [10] and health disparities [11]. In HF has not been closely integrated with research on comorbidities in HF. Because HF research is often confined to silos, little is known about interrelationships among socioeconomic factors, disparities, and comorbidities in HF.

HF may be only one of several difficult challenges for patients with multimorbidity, stressful socioeconomic circumstances, and psychosocial problems [12]. Unfortunately, the fragmentation of research on HF has left us with little understanding of patients with such varied and complex health needs. The purpose of this study is to identify disparities and patterns of comorbidity that may affect the course of HF and that may require different mixtures of medical, psychological, and social services to address. It will encompass both psychiatric and medical comorbidities but will emphasize psychiatric comorbidities.

*Syndemics theory* is a conceptual framework for studying this type of psychosocial and medical complexity. Syndemics, also known as synergistic epidemics, occur when there are adverse interactions between coprevalent disorders. Syndemic disorders tend to concentrate in populations that are made vulnerable by social determinants of health (SDOH) [13]. The syndemics framework has yielded important insights into other disorders such as HIV/AIDS, but it has not been applied to the complex problems of patients with HF. The *multimorbidity* construct provides an alternative approach for investigating the effects of multiple comorbidities on health-related outcomes. This study will be the first to use both the syndemics and multimorbidity frameworks to examine the effects of SDOH, psychiatric comorbidities, and interactions between them, on readmissions, self-care, and global health in patients with HF.

## SIGNIFICANCE

### Background

This project addresses the psychosocial and medical complexity of patients with HF. This is significant because socioeconomic disadvantages, health disparities, and multimorbidity complicate the course, treatment, and cost of HF and other chronic illnesses [14]. There has been little if any research on the syndemics of HF comorbidities, but there have been numerous studies of HF risk factors, comorbidities, and vulnerability factors. The following review focuses on studies that are relevant to this project.

### Race and Sex Disparities

Black men have worse HF outcomes than other groups defined by race and sex. A study of over 55 million Medicare beneficiaries found higher age-adjusted HF hospitalization rates in black men than in other groups [15]. This was recently replicated in a study of 1.7 million hospital stays in the US National Inpatient Sample [16]. Racial disparities in HF outcomes are present even among insured patients [17,18].

### Socioeconomic Disparities

Socioeconomic status (SES) affects HF outcomes. In a national sample of Medicare beneficiaries (*n* = 25,086) hospitalized with HF, patients in the lowest SES category had a higher risk of readmission and mortality within one year of discharge [19]. Among 1342 participants in the Atherosclerosis Risk in Communities (ARIC) study with an initial HF hospitalization and a high level of comorbidity, those who lived in low-income neighborhoods had a higher risk of rehospitalization or death compared to those in higher-income neighborhoods [20]. Among 2331 participants in the Heart Failure: A Controlled Trial Investigating Outcomes of Exercise Training (HF-ACTION) trial, low income was associated with worse functional capacity and quality of life, and it predicted all-cause hospitalization and death over 2.5 years in a univariate analysis but was not significant in an adjusted model [21]. A recent prospective study of 1802 patients with HF, a high score on the UK. Index of Multiple Deprivation (IMD) predicted all-cause hospitalization and mortality and a longer cumulative duration of hospitalization over one year [22]. Socioeconomic disadvantage is also associated with higher rates of cardiovascular health risk behaviors such as smoking [23].

Whether a patient has health insurance and if so, which type, affects HF outcomes. In a study of 4584 HF index admissions, the adjusted odds of all-cause readmissions were 32% higher, and those of HF readmissions were 68% higher, among Medicaid than commercially insured patients [24]. A study of 558,904 patients with HF in the California Patient Discharge Database compared 30-day readmission rates before vs after the federal Hospital Readmissions Reduction Program (HRRP). The readmission rate dropped among Medicare patients but not among patients with commercial insurance [25]. A 2013 study of 60,814 patients in the National Cardiovascular Data Registry found that 9.4% were uninsured, 19.7% had public insurance, and 70.9% were privately insured. Uninsured patients were younger, predominantly female, more likely to have HF, and less likely to be treated with guideline-recommended medications than other patients [26].

### Psychiatric Comorbidities

#### Depression

Approximately 20% of patients with HF have comorbid major depression MD [27], but we found that in 682 patients with HF, the prevalence of MD differed by age and New York Heart Association (NYHA) class; it was 67% in younger patients in Class IV (i.e., severe HF symptoms and functional impairment) [28]. We also found that MD during the index hospitalization predicted multiple readmissions over one year [29] and decreased survival [9]. Other studies have also found that depression increases the risk of hospitalization [30,31] and decreases survival [30–37] in HF. Depression is also associated with poor physical [38–40] and neurocognitive [41] functioning, medication nonadherence[42,43], and poor HF self-care [44,45].

#### Anxiety

We conducted a retrospective cohort study (*n* = 236,079) of Veteran’s Administration (VA) patients who were free of cardiovascular disease at baseline. Patients with an anxiety disorder, MD, or both were at higher risk of incident HF compared to those with neither disorder [46]. Anxiety may also have prognostic value in established HF [47,48], although some studies have found that it does not have independent prognostic value after adjusting for depression [36,49]. Similarly, anxiety is negatively associated with HF self-care but depression appears to have a stronger effect [50].

#### Posttraumatic stress disorder (PTSD)

Several studies of trauma-exposed populations have found increased odds of incident cardiovascular disorders including HF. A prospective study of 8248 VA patients found that PTSD increased the risk of HF [51]. A retrospective study of 251 low-income, predominantly black HF patients also found that PTSD was associated with an earlier onset of HF [52]. PTSD was also associated with HF in the Canadian Community Health Survey (*n* = 36,984) [53] as well as in a German community-based study (*n* = 3171) [54]. In contrast, little is known about PTSD in patients with established HF.

#### Psychotic disorders

Few studies have examined associations between psychotic disorders and HF outcomes. In a study of 611 black patients hospitalized with HF, 7% had schizophrenia and 2% had bipolar disorder. Both conditions were associated with a higher risk of 30-day readmission; neither predicted a higher risk of mortality [55]. A study of 255 elderly patients with serious mental illnesses and 533 nonaffected primary care patients found higher rates of emergency department visits and longer lengths of stay for medical hospitalizations in those with schizophrenia or bipolar disorder even though the prevalence of HF and other major medical illnesses did not differ [56]. An 11.5-year follow-up of the Baltimore Epidemiologic Catchment Area Study found that participants with bipolar disorder had an increased risk of myocardial infarction or HF [57]. A more recent Danish population-based cohort study including 36,718 hospitalized with incident HF found that patients with schizophrenia had a higher risk of one-year mortality but no higher risk of rehospitalization [58].

#### Substance use disorders

Substance use disorders are prevalent comorbidities in HF. Some of them contribute to precursors of HF such as hypertension and coronary heart disease and/or to cardiomyopathies. Several substance use disorders paradoxically predict better short-term HF outcomes, probably because they accelerate the development of HF and thus are inversely associated with age in patients with HF.

Several studies have shown that smoking is a risk factor or that smoking cessation is a protective factor for incident HF [59,60]. The OPTIMIZE-HF trial produced the first large-scale analysis of the effects of smoking in established HF. Out of 48,612 patients, 7743 (16%) were smokers. Paradoxically, smokers had a lower mortality risk than nonsmokers, probably because they were younger [61]. However, in the Survival and Ventricular Enlargement (SAVE) trial (*n* = 2231), smoking cessation predicted a 40% lower risk of all-cause mortality and a 30% lower risk of myocardial infarction (MI), HF hospitalization, or death [62]. Smoking also predicts mortality in HF patients on heart transplant lists [63]. A recent analysis of the National Inpatient Sample found a 15.5% prevalence of tobacco use disorder in patients hospitalized with HF [64].

Although alcoholic cardiomyopathy has been recognized for decades as a cause of HF [65], heavy alcohol consumption was not associated with an increased risk for HF in the Framingham Heart Study [66]. There has been little research on the prevalence or effects of alcohol use disorder in established HF.

Stimulants contribute to the development and progression of HF [67,68]. A study of 11,258 patients with HF in the Acute Decompensated Heart Failure National Registry Emergency Module found that 5.3% of patients reported current or past stimulant use, and that stimulant use was associated with worse left ventricular dysfunction [69]. A study of 590 outpatients with cardiomyopathy or HF at a regional health center found that 38% used methamphetamine, and methamphetamine use was associated with worse left ventricular dysfunction [70]. A retrospective study of 141 patients hospitalized with HF in California found that methamphetamine use was associated with a worse NYHA class [71]. A recent study of 267 patients with HF, 90 of whom used cocaine, found that the cocaine users had a lower risk of major adverse cardiovascular events (MACE) than nonusers, probably because the cocaine users were 10 years younger on average [72].

Among 10 million patients with HF in the Nationwide Inpatient Sample, 29,014 had opioid use disorder. Opioid use was associated with younger age and lower risk of in-hospital mortality [73].

### Medical Comorbidities

Common medical comorbidities in HF include hypertension, coronary artery disease (CAD), cardiac arrhythmias, anemia, diabetes, chronic obstructive pulmonary disease (COPD), obstructive sleep apnea (OSA), osteoarthritis, and renal insufficiency [74]. HF is also associated with dementia. Associations between HF and Alzheimer’s disease have been found in large cohort studies [75–78]. Associations with other dementias have also been found [77,78]. Both medical and psychiatric comorbidities complicate the treatment of HF [5] and increase the risks of readmission [20,79–81] and death [20,82,83].

### Multimorbidity

**Figure 1.**
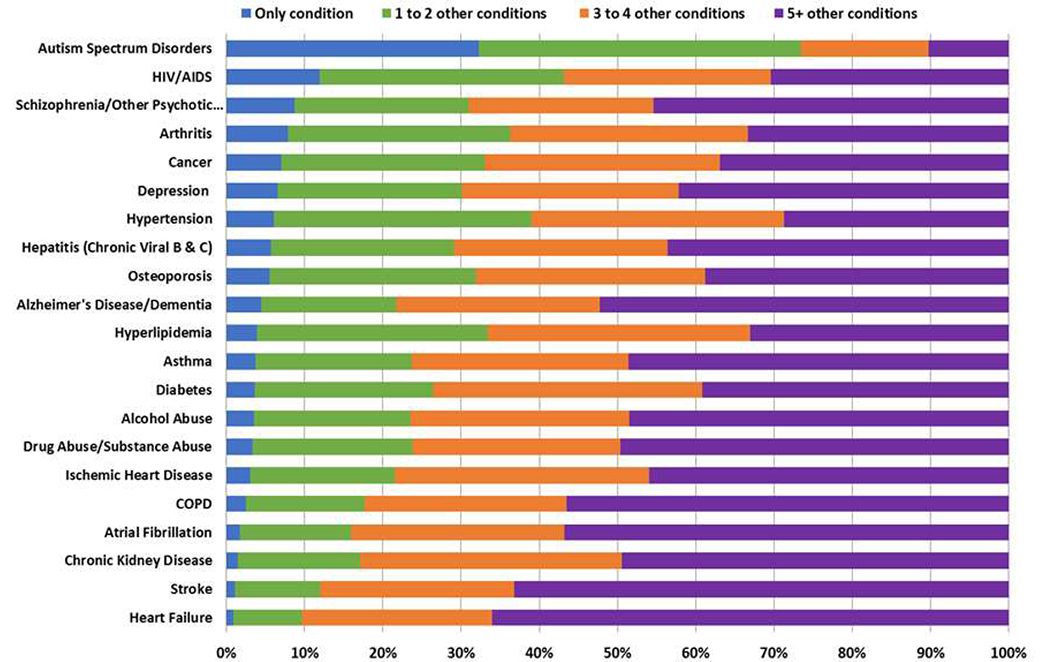
Comorbidity among chronic conditions for Medicate Fee-for-Service beneficiaries (2017) [87].

**Figure 2.**
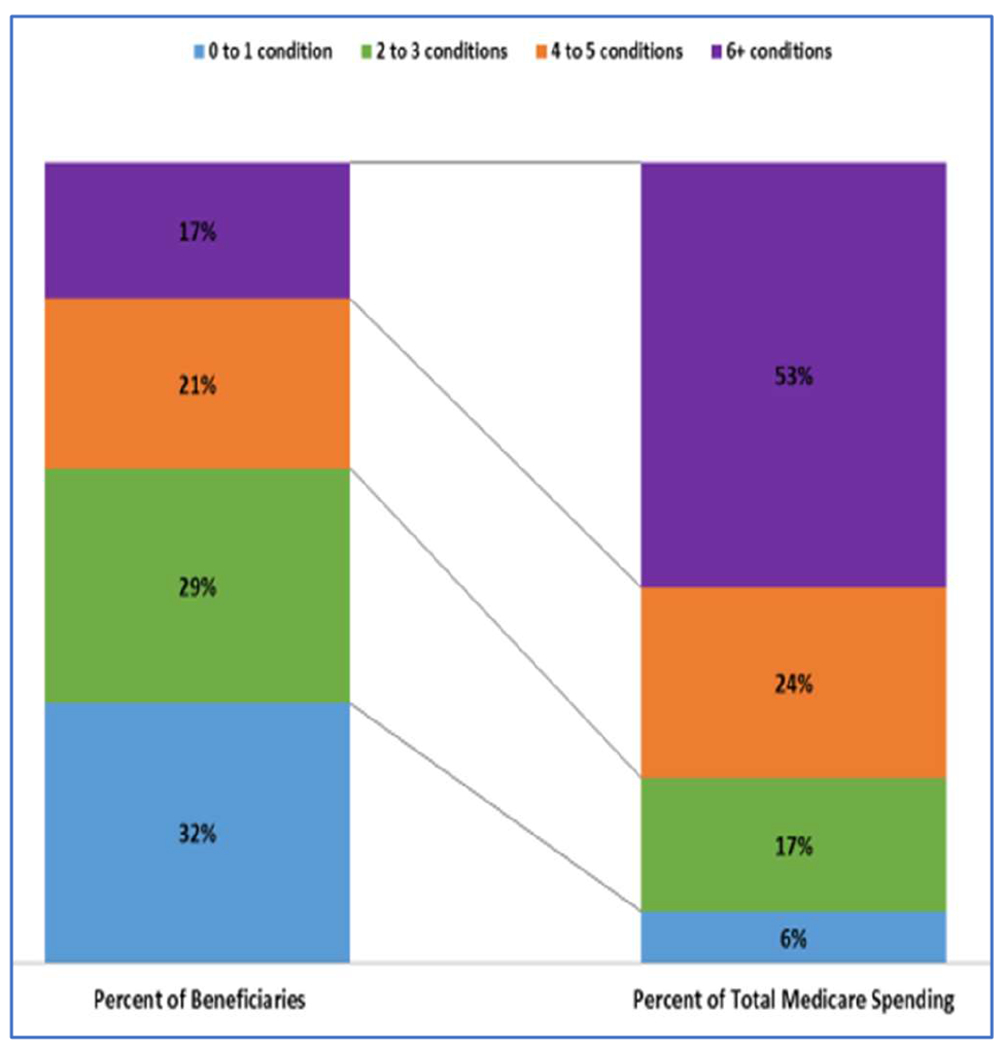
Distribution of Medicare Fee-for-Service beneficiaries and Medicare spending by number of chronic conditions (2017) [87].

**Figure 3.**
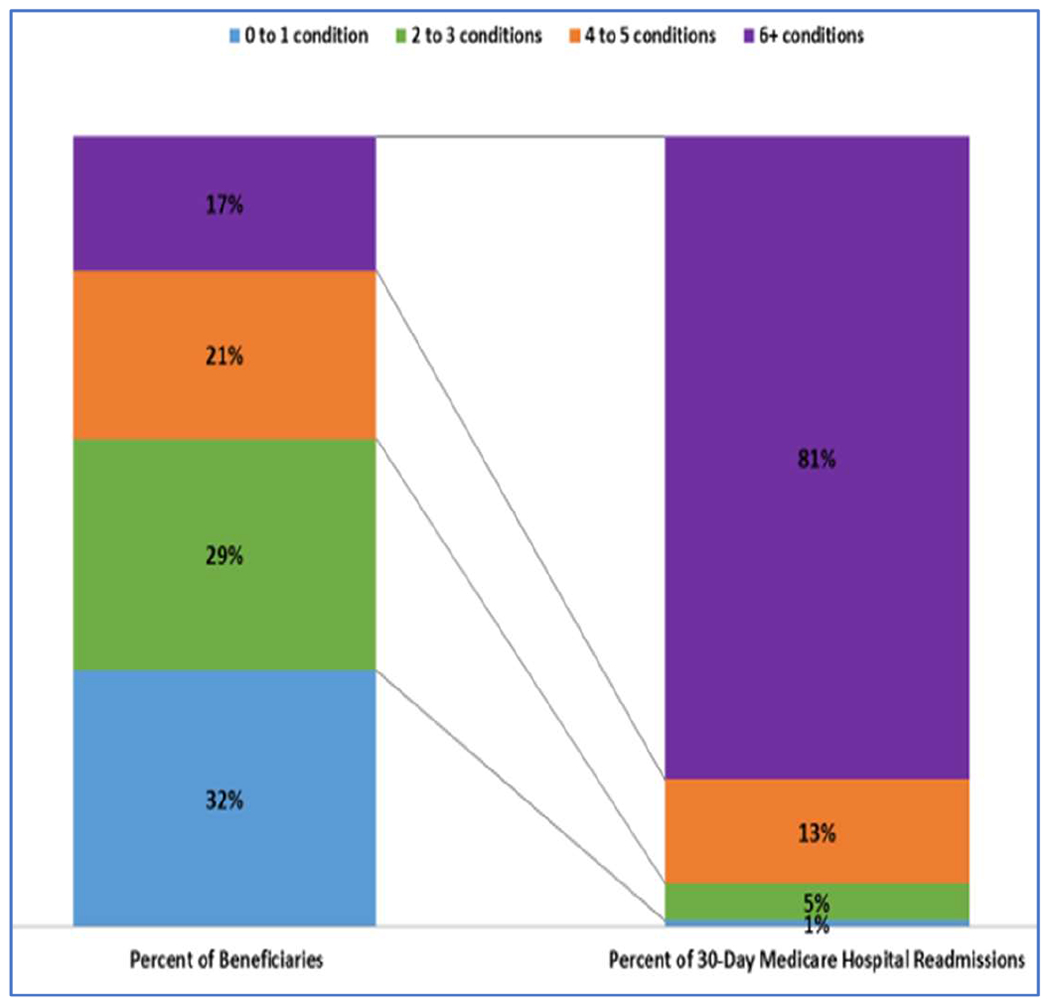
Distribution of Medicare Fee-for-Service beneficiaries and 30-day Medicare hospital readmissions by number of chronic conditions (2017) [87].

Multimorbidity is common in HF [6,84,85]. Older patients tend to have more comorbidities than younger patients, although the prevalence of comorbidities is increasing among younger patients [86]. In 122,630 Medicare beneficiaries with HF, 40% had ≥5 noncardiac comorbidities, often in conjunction with one or more cardiac comorbidities [74]. According to the latest (2017) data from CMS, 29% of Medicare beneficiaries have 2–3 chronic conditions, 21% have 4–5, and 17% have 6 or more [87]. Figure 1 displays the latest CMS data on numbers of comorbid conditions in a variety of index conditions. More patients with HF have ≥5 comorbidities than patients with any other index condition, and very few cases of HF present with no comorbidities. Figures 2 and 3 illustrate the dramatic effects that multimorbidity has on Medicare spending and 30-day readmissions, especially in patients with a total of ≥6 chronic conditions.

Like most multimorbidity research, the CMS analyses use a simple *count* of chronic conditions to measure the level of multimorbidity. The count in any given study depends on which (and how many) disorders are counted. This differs across studies and areas of research. In the proposed study, the comorbidities that will be examined in the syndemics analyses will also comprise the multimorbidity count.

An implicit assumption of multimorbidity counts is that comorbidities have *additive* rather than synergistic effects. Figures 2 and 3 suggest that linear additivity may be an oversimplification, in that outcomes are much worse for patients with ≥6 conditions than for those with fewer. All participants in our study will have HF, and the presence of HF counts toward the overall burden of multimorbidity. Consequently, the cut point of interest in our study is ≥5 comorbidities (not 6).

### INNOVATION

This study will be the first to apply the syndemics framework to HF research and one of the first tests of syndemics theory in any area of behavioral medicine other than HIV/AIDS. It will serve as an example of the potential of the syndemics framework to advance our understanding of the adverse effects of health disparities and of psychosocial and medical multimorbidity and will help to encourage further syndemics research in cardiovascular behavioral medicine and in other areas of behavioral medicine.

The syndemics concept originated in the 1990’s, in the medical anthropology literatures on substance abuse [88] and HIV/AIDS [89]. It was initially used to investigate the convergence of these interrelated epidemics in impoverished inner-city communities and in populations that experience discrimination, stigma, abuse, or other forms of victimization. It is still being applied to these areas of research, but syndemics theory is also playing a growing role in other areas of epidemiological and clinical research [13].

In a syndemic (synergistic epidemic), there are adverse interactions between two coprevalent disorders that may increase the severity of one or both. For example, HIV/AIDS and tuberculosis (TB) interact to cause excess morbidity and mortality. AIDS increases the risk of incident TB, infection with HIV predicts progression from TB infection to TB disease, and TB is a frequent cause of death in HIV/AIDS. Both conditions require complex medication regimens and pose high risks of nonadherence and drug-drug interactions [90].

Syndemics theory also addresses the socioeconomic contexts of coprevalent disorders. It predicts that they cluster within populations that are vulnerable due to poverty, discrimination, lack of access to health care, or other adverse social or socioeconomic circumstances [91]. Thus, investigations of syndemics ask four interrelated questions: (1) Are the disorders coprevalent? (2) Are there any adverse interactions between them? (3) Do they cluster within vulnerable subpopulations? and (4) Do these disease clusters help to explain why the vulnerable subpopulations have worse health outcomes? The first and second questions are typically addressed in the entire population of interest, which in our case consists of patients with HF. When interacting disorders are found, then the third and fourth questions are asked.

Regardless of whether two coprevalent disorders are more severe when they occur jointly rather than alone, interactions between them may complicate *other* disorders with adverse effects that are worse than additive. When the syndemics framework is used to study comorbidities in a focal disorder such as HF, the conditions of interest must be coprevalent not only with each other, but also with the focal disorder. In addition, the “adverse interaction” questions concern potential impacts on the focal disorder or on global health outcomes rather than on the severity of the interacting comorbidities themselves. We may find, for example, that depression and diabetes are coprevalent comorbidities in patients with HF. Whether depression and/or diabetes are more severe in patients with both comorbidities than in those with only one is not the key question. The more important question is whether patients with both comorbidities have *worse HF outcomes* than would be expected if depression and diabetes had independent or additive effects.

It is important to note that adverse interactions and clustering within vulnerable subgroups are *hypotheses* based on syndemics theory, not foregone conclusions. We may find that some coprevalent comorbidities do *not* interact, or that some comorbidities that *do* interact *do not* cluster within vulnerable subgroups. We may also find that multimorbidity is a more parsimonious explanation than syndemics for adverse HF outcomes. However, so little is known about the impact of psychiatric comorbidities other than depression in HF that this study will yield important new information about them even if none are syndemic. We know little, for example, about the characteristics of patients with HF who have comorbid substance use disorders. Thus, the study will yield reportable results that extend beyond our primary aims.

## SPECIFIC AIMS

### Modifications of Aims

We originally proposed to recruit, enroll, and assess patients with HF during a hospital stay, and to examine 30-day readmissions as the primary outcome. However, the COVID-19 pandemic started to disrupt research and clinical operations at our medical center during the start-up phase of the study. One of the consequences of this development was that it became impossible to conduct this type of research with hospitalized patients. Consequently, we revised the protocol and obtained approval for the modified research plan from the National Heart, Lung, and Blood Institute (NHLBI) and our institutional review board.

The modified plan is to use the hospital’s electronic medical record (EMR) system to identify study candidates, recruit them within 30 days after discharge via mail and telephone, and conduct the baseline assessments via telephone. Because the baseline assessments will be conducted during rather than before the 30-day post-discharge period, it is no longer feasible to prospectively study 30-day readmissions. Instead, we will study multiple readmissions over the entire follow-up phase. We have also made some adjustments to the baseline assessment battery to fit these unanticipated circumstances. For example, we will use a different dementia screener than originally planned; the Short Blessed Test (SBT) can be administered over the telephone but the instrument we originally planned to use is not suitable for telephone administration.

Also, the shift to remote recruitment and assessment creates the possibility of recruiting patients from other hospitals. Initially, recruitment will be restricted to one hospital, as originally planned. If we receive permission from other hospitals, and if it is logistically feasible to expand recruitment, we will do so.

### Specific Aims (Revised)

1. To determine the coprevalence of psychiatric and medical comorbidities in patients with HF.
2. To test the hypotheses that coprevalent comorbidities have synergistic effects on:

- All-cause readmissions and mortality risk,
- HF self-care, and
- perceived health.
- To identify vulnerable subpopulations of patients with HF who have higher coprevalences of syndemic comorbidities compared to less vulnerable subpopulations.
- To determine the extent to which syndemic comorbidities explain adverse outcomes (readmissions and mortality, poor HF self-care, and poor perceived health) in vulnerable subgroups of patients with HF.
- To determine the effects of multimorbidity on readmissions, mortality, self-care, and perceived health.

## METHODS

### Eligibility Criteria

#### HF Inclusion criteria

A daily query of the Epic EMR system at Barnes-Jewish Hospital at Washington University Medical Center in St. Louis will identify patients who meet the study’s HF inclusion criteria, which require that the patient (1) has a documented clinical diagnosis of HF and (2) meets the European Society of Cardiology (ESC) 2016 “signs and symptoms” criteria for the diagnosis of HF on admission or during the hospital stay [92]. The ESC criterion requires the presence of ≥2 typical symptoms and/or specific signs of HF. The typical symptoms of HF include breathlessness, orthopnea, paroxysmal nocturnal dyspnea, reduced exercise tolerance, fatigue, and ankle swelling. The specific signs of HF include elevated jugular venous pressure, hepatojugular reflux, third heart sound, and laterally displaced apical impulse. Patients who meet these criteria will also meet the American College of Cardiology Foundation (ACCF)/American Heart Association (AHA) criteria for Stage C or D heart failure. In addition, the eligibility criteria include patients with preserved (HFpEF), mid-range (HFmrEF), and reduced (HFrEF) ejection fraction. However, the objective is to enroll patients with chronic HF. Consequently, cases of isolated right HF (cor pulmonale) or reversible HF due to valve disease with impending surgical correction do not fit the study’s eligibility criteria. Recruitment will be expanded to other hospitals in the St. Louis area if and when feasible.

#### Exclusion criteria

Patients who meet any of the following exclusion criteria will not be eligible to participate in the study: (1) Patient’s physician opposes study participation; (2) patient or legally authorized representative (LAR) refuses to participate; (3) patient is too ill to participate; (4) logistical or communication barrier; (5) unavailable to participate within 30 days of discharge from the index hospitalization.

### Recruitment, Consent, and Assent

#### Patients with clinically diagnosed dementia

If a patient who meets the inclusion criteria has a diagnosis of any type of dementia or other condition such as stroke or head injury that has caused severe cognitive impairment, the study recruiter will contact the patient’s LAR by mail and telephone, explain the study, and ask the LAR to consent to the patient’s participation. The patient’s verbal assent will be sought if the patient is able to cooperate. Patients with dementia or other conditions that have caused severe cognitive impairment will be exempted from the baseline interviews and questionnaires; the only data to be obtained will be extracted from the EMR.

#### Patients without clinically diagnosed dementia

If a patient who meets the HF inclusion criteria does not have a clinical diagnosis of dementia or other documented condition that has caused severe cognitive impairment, the study recruiter will contact the patient by mail and telephone, explain the study, and seek the patient’s consent to participate.

If there is any uncertainty as to whether the patient is sufficiently cognitively intact to complete the interviews and questionnaires, the recruiter will administer the Short Blessed Test (SBT) [93]. Patients who score ≥16 on the SBT will be exempted from the interviews and questionnaires due to cognitive impairment; the only data to be obtained will be extracted from the EMR.

### Baseline Medical Data

Baseline medical data will be obtained from the EMR documentation of the index hospitalization and recorded on the Baseline Medical Form. If the patient is cognitively intact, the interviewer will review the medical data with the participant and use self-reported data to augment the EMR data.

The initial diagnosis of HF will be classified as within the past year or more than one year ago. NYHA class for the past two weeks will be determined by interview if possible or estimated from the EMR if necessary. The Rockwood et al. Frailty scale [94] will be used to rate the patient’s frailty level, with scores ranging from 1 (very fit) to 7 (severely frail). The rating will be based on a review of the EMR data and the baseline assessments. The comorbidities and procedures listed in Table 1 will be documented.

When available, the following lab values will be obtained from the index hospitalization record: Brain natriuretic peptide (BNP), N-terminal pro-brain natriuretic peptide (NT-proBNP), blood urea nitrogen (BUN), creatinine, estimated glomerular filtration rate (eGFR), and hemoglobin. If a test is repeated during the hospital stay, only the initial value will be recorded.

The index hospitalization record will be examined to determine whether a toxicology screen was performed during this hospitalization or in the emergency department. The presence or absence of substances listed in Table 2 will be noted.

Echocardiographic data will also be obtained from the EMR. Echocardiography findings the index hospitalization will be obtained if available; if not, the most recent echocardiogram performed within the past year will be used. Data to be recorded, when available, include left ventricular ejection fraction (LVEF), pulmonary artery systolic pressure, valve dysfunction, diastolic dysfunction, right ventricular dysfunction.

### Baseline Psychosocial and Functional Data

#### Social determinants of health

Various instruments for documenting SDOH have been used in recent studies (e.g., [95–97]). Because a well-validated and widely-used instrument was not available, we developed the Heart Failure Social Determinants of Health (HF-SDOH) form (included in the Supplement). The items are based on ICD-10 “Z” codes and items assessed in previous studies. It is divided into the following sections: (1) Demographics, (2) Education, (3) Employment and Unemployment, (4) Income and Savings, (5) Problems Related to Housing and Economic Circumstances, (6) Problems Related to Social Environment or Upbringing, Problems Related to Family and/or Primary Support Group, (7) Legal and Social Problems, and (8) Problems Related to Health Care.

Many items are coded in terms of whether the problem was present during the following time frames: (1) within the past 2 months, (2) within the past 2 years, (3) more than 2 years ago. The “past 2 months” code is intended to reflect problems that are currently present or that have occurred in the recent past. The “within past 2 years” code covers problems that occurred between about 2 and 24 months ago. Thus, both, neither, or just one of the 2-month and 2-year codes may be checked for a given problem. For example, if a patient became homeless for the first time within the past month, “within past 2 months” would be checked, but “within past 2 years” would not be checked. Conversely, if the patient was homeless around a year ago but currently has stable housing, “within past 2 years” would be checked but “within past 2 months” would not be checked. Both would be checked if the patient is currently homeless and has been for a year or two.

The HF-SDOH is administered as a semi-structured interview. The interviewer asks a general but tailored question for each section and follows up as needed with more specific probes. For example, the interview starts with Employment and Unemployment section with a general question about current employment status. If the patient reports being retired, additional questions about work-related problems will be asked in terms of difficulties that may have occurred in the past, e.g., having to work multiple jobs. In contrast, the questioning would cover both past and present for a currently employed patient.

The HF-SDOH asks about current stressors but not about perceived stress. The 10-item Perceived Stress Scale (PSS) [98] was added to the protocol in response to reviewers’ recommendations. This change was implemented in April 2021, after enrollment had already started.

#### Psychiatric comorbidities

After completing the HF-SDOH interview, the interviewer will administer selected modules from the NETSCID-5 interview [99] to assess psychiatric disorders according to the criteria of the 5th edition of the American Psychiatric Association Diagnostic and Statistical Manual (DSM-5) [100]. The disorders to be assessed were found in previous studies to be prevalent in HF or coronary heart disease, are associated with the development of HF, and/or are known to complicate HF. Table 3 lists the disorders of interest.

#### Heart failure self-care

The revised Self-Care of Heart Failure Index (SCHFI v7.2) [101] will be administered to assess the quality of HF self-care during the past month. The SCHFI is well validated and is one of the most widely-used measures of heart failure self-care. It consists of 29 items divided into 3 scales that measure (1) self-care maintenance, (2) symptom perception, and (3) self-care management. Higher scores reflect better self-care.

The Maintenance scale assesses behaviors that patient with HF use to maintain health or prevent new or worsening medical problems, such as engaging in exercise, adhering to a low-salt diet, and taking medications as prescribed. The Symptom Perception scale assess changes that patients with HF commonly monitor, such as weight, ankle swelling, and shortness of breath on exertion. The Management scale assesses behaviors that patients with HF often use to control or respond to symptoms, such as reducing salt and fluid intake or calling a healthcare provider for guidance.

#### PROMIS measures

Three Patient-Reported Outcomes Measurement Information System (PROMIS) short-form questionnaires will also be administered at baseline. First, the 10-item PROMIS Global Health v1.2 short form [102] will be administered to assess global perceived physical, mental, and social health and health-related quality of life. This instrument will provide the data for the global perceived health outcome.

Second, the 4-item PROMIS Self-Efficacy for Managing Medications and Treatment short form [103] will be used to assess the patient’s confidence in his or her ability to manage medications. The data from this instrument will provide some additional information about self-care that is not captured on the SCHFI.

Third, 8-item the PROMIS Self-Efficacy for Managing Daily Activities short form [103] will be used to assess instrumental activities of daily living. The data from this instrument will provide information about functional impairment that will complement the NYHA and frailty ratings.

### Follow-up Data

#### Patient readmissions

One of the revised aims of the study is to examine the effects of SDOH and psychiatric comorbidities on multiple readmissions. We will use the EMR to identify the dates of admission and discharge for every readmission to Barnes-Jewish Hospital or to affiliated hospitals during throughout the follow-up phase of the project. We will also ask patients who receive care at Veterans Administration facilities to sign a release of information for to enable us to obtain records of admissions to these facilities.

The limitation of this approach is that we will not be able to identify and document all readmissions to hospitals that are not affiliated with Barnes-Jewish Hospital or with the Veterans Administration. In previous studies, we contacted patients on a regular schedule during the follow-up phase and asked them to identify any readmissions that have occurred during the interval since the previous contact. We then asked them to sign release of information forms so that we could obtain records of these readmissions.

That approach will not be feasible in this study because the eligibility criteria are very broad. As a result, we expect the cohort to include patients who are homeless, who have unstable housing arrangements, and/or whose psychosocial problems (e.g., alcohol or substance abuse) will make them difficult to contact on a regular basis. Thus, we may not be able to ascertain 100% of the readmissions. In our experience, however, most readmissions of patients with HF occur at the same hospital as the index admission. Consequently, we expect to capture most of the readmissions.

#### Mortality

The EMR will be used to document the dates of deaths that occur during readmissions to Barnes-Jewish Hospital, affiliated hospitals, or Veterans Administration hospitals. We will search online obituaries and the Social Security Death Index to document deaths that occur outside of these facilities. If feasible, we will also conduct a search of the National Death Index.

## STATISTICAL ANALYSIS PLAN

### Data Management

The Research Electronic Data Capture (REDCap) system [104] will be used for HIPAA-compliant data entry and data management. NetSCID-5 data on stored on a cloud server. The REDCap and NetSCID-5 data will be exported to SAS© 9.4 datasets (SAS Institute, Inc., Cary NC, USA) for analyses and quality control reports, including evaluation of model variable distributions, identification of outliers and invalid values, and examination of missing data patterns to build efficient multiple imputation models.

### Attrition

Patients with dementia cannot provide reliable responses to interview questions, and dementia is a rule out criterion for the DSM-5 disorders of interest. Thus, the psychiatric disorders will be coded “absent” if the patient has dementia, and the self-management and global health scores will be estimated from the patient’s clinical status. Thus, there will effectively be no loss of self-report data in this subgroup.

For participants without dementia, the patient’s direct involvement in the study will be limited to the baseline interview and questionnaires. These assessments might be interrupted in some cases, e.g., by precipitous changes in medical or cognitive status. Based on our previous experience with research on patients with HF, we estimate that approximately 5% of the participants without dementia will be unable to complete the assessments. Thus, we expect to obtain complete data on ***n*** = **508** patients and will use multiple imputation for data that are likely missing at random (MAR).

### Planned Statistical Analyses

#### Aim 1. Coprevalence analyses

We will use one-way Chi-square goodness-of-fit tests to identify comorbidities for which the coprevalence is significantly higher than would be expected by chance alone. These analyses will be limited to conditions that occur in >5% of the sample.

#### Aim 2. Synergistic effects of coprevalent conditions

The coprevalences of comorbid conditions (even the most common ones) are likely to be too low to support adequately powered interaction tests. As an alternative, we will create ordinal **syndemic exposure factors (SEFs)** representing the number of conditions present in each comorbidity pair (0, 1, or 2). The relationship between the SEF and the outcome of interest can be linear or curvilinear; the latter is more consistent with a synergistic effect. The primary analyses will focus on psychiatric-medical comorbidities and pairs of psychiatric comorbidities; additional analyses will test syndemic relationships between pairs of medical comorbidities.

#### Aim 2a (Revised). Multiple All-Cause Readmissions:

We will fit separate marginal proportional rates models [105] to each pair of coprevalent conditions, to test the hypothesis that higher SEF scores predict more all-cause hospital readmissions, after adjusting for covariates that predicted multiple all-cause readmissions in a previous study (Freedland et al., under review), including age, New York Heart Association (NYHA) class, diabetes, chronic obstructive pulmonary disease (COPD), hypertension, and estimated glomerular filtration rate (eGFR). For any given model, the factors that comprise the SEF will be excluded from the covariate list. For example, COPD will be removed from the list of covariates in a model in which the SEF refers to the combination of depression and COPD. Additional covariables that are needed for Aim 3 (below) will be added.

#### Aim 2b. (Revised). Self-Care of Heart Failure:

We will regress the Maintenance, Management, and Confidence scores from the Self-Care of Heart Failure Index (SCHFI v7.2) on each SEF in one-way analysis of covariance (ANCOVA) models, adjusting for known correlates of HF self-care including age, race, education, number of medications, and smoking status. Additional covariables that are needed for Aim 3 (below) will be included. Statistical inference will be based on the planned model contrasts: C_1_ = μ0 – μ1, C_2_ = μ0 – μ2, and C_3_ = μ1 – μ2, where μ*i* represents the mean response within level i of the SEF.

#### Aim 2c. Perceived Global Health:

We will regress the 12-item PROMIS Global Health short form V1.2 on each SEF in one-way analysis of covariance (ANCOVA) models, adjusting for known correlates of perceived health in patients with HF including NYHA class, race, and perceived sufficiency of income [106]. Additional covariables that are needed for Aim 3 (below) will be included.

### Aim 3. Vulnerable subgroups

Vulnerable subgroups will be defined by race, sex, and social determinants of health (SDOH). Examples of SDOHs include financial difficulties, limited access to health care services, and exposure to violence.

#### Aim 3a. Effects of Race, Sex, and SDOH on Outcomes:

Vulnerable subgroups of patients with HF will be examined in the same models that will be tested for Aim 2.

#### Aim 3b. Clustering of Syndemic Conditions within Vulnerable Subgroups:

We will fit proportional odds models to test the relationship of each vulnerability factor (e.g., race) to each SEF. The odds ratio associated with the vulnerability factor (OR_vf_) is the effect of interest.

### Aim 4. Effects of syndemic clusters on outcome disparities in vulnerable subgroups

We will add SEFs to the Aim 3a models. If adding the SEF attenuates the effect of the vulnerability factor on the outcome, this supports the hypothesis that the syndemic pair partially accounts for the effect of the vulnerability factor on the outcome.

### Aim 5. Effects of multimorbidity on outcomes

This aim focuses on the effects of the overall burden of multimorbidity on hospital readmissions, HF self-care, and perceived global health. The Centers for Medicare & Medicaid Services (CMS) has found that costs and readmissions are dramatically higher in patients with ≥6 chronic conditions than patients who have <6 conditions [87]. Since all participants in this study will have heart failure, we will reduce the multimorbidity cut point by one to ≥5 (vs <5) comorbidities. This categorical variable will be used in the primary analyses because of the CMS findings and because of the skewness of multimorbidity counts. The raw counts will be used in secondary analyses, along with the Charlson and Deyo weighted indices.

### Aim 5a. Overall Effects of Multimorbidity:

We will use a marginal proportional rates model to test the hypothesis that multimorbidity is an independent predictor of hospital readmissions, after controlling for known predictors including age and NYHA class and excluding comorbidities. We will use one-way ANCOVAS to examine the effects of multimorbidity on the HF self-care and global health outcomes.

### Aim 5b. Differences in Multimorbidity between Vulnerable Subgroups:

We will use logistic regression models to examine the effect of multimorbidity on the dichotomous vulnerabilities discussed in Aim 3a.

### Aim 5c. Differences in Multimorbidity as Explanations for Outcome Disparities:

We will use the same analytic approach described in Aim 4 except that the SEF will be replaced by multimorbidity category.

### Exploratory aims

Logistic regression will be used to analyze associations between prevalence SDOHs and prevalent psychiatric comorbidities. The Liu et al. model will be used to analyze the relationship between HL self-care and readmissions [105].

### LIMITATIONS

The sample will consist of volunteers, so it may not be fully representative of the HF population. The baseline assessments will not be repeated, so it will not be possible to detect changes in SDOH or psychiatric comorbidities or determine how these changes affect HF outcomes. Readmissions that are not documented in the EMR or that do not occur at Veterans Administration facilities will not be ascertained.

## DISCUSSION

This observational cohort study will be one of the first to examine relationships between social determinants of health and psychiatric comorbidities in patients with heart failure, and to investigate their effects on multiple readmissions, HF self-care, and global health. It will be one of the first to apply the syndemics framework to research on HF, and to determine whether the multimorbidity framework offers a more parsimonious approach than the syndemics framework to predicting HF outcomes. Many of the SDOH factors and psychiatric comorbidities have not been studied before in patients with HF.

Since this study is not a trial, the results may not have direct clinical or public health implications. However, an overarching goal of this project is to identify problems, or clusters of problems, that are prevalent in patients with HF, that affect outcomes, and that are not routinely addressed in HF care. Identification of these problems is a prerequisite for the developing and testing of interventions designed to treat them. Additional studies will be needed for these purposes.

## Figures and Tables

**Table 1.** Medical comorbidities and procedures to be documented based on EMR data at index.

Condition or Procedure	Coding^1^
Hypertension	Absent/present
Hyperlipidemia	Absent/present
History of tobacco smoking or vaping	Never, current, past
Coronary artery disease	Absent/present
History of MI or unstable angina	Absent/present (past and/or index)
Cardiomyopathy (amyloid, dilated, drug or alcohol-related, hypertrophic, ischemic, nonischemic, restrictive, other)	Absent/present
Atrial fibrillation or flutter	Absent/present
History of infective endocarditis	Absent/present
Pacemaker	Absent/present
Automatic implantable cardioverter defibrillator (AICD)	Absent/present; ever fired yes/no
History of percutaneous coronary intervention (PCI)	Absent/present (past and/or index)
History of coronary artery bypass graft (CABG) surgery	Absent/present (past and/or index)
Valve disease (≥moderate, not tricuspid)	Absent/present
Valve surgery	Absent/present
Left ventricular assist device (LVAD)	Absent/present
Pulmonary hypertension	Absent/present
Chronic obstructive pulmonary disease	Absent/present
Asthma	Absent/present
Sleep apnea	Absent/present
Cerebrovascular accident (stroke)	Absent/present
Diabetes	Absent/present
Autoimmune disease	Absent/present
Peripheral artery disease	Absent/present
Venous disease	Absent/present
Osteoarthritis	Absent/present
Malignancy in past 5 years (excluding basal cell)	Absent/present
Thyroid disease	Absent/present
Chronic kidney disease stage ≥3	Absent/present
Cirrhosis	Absent/present
Positive COVID test (ever)	Absent/present
Obesity	Absent/present

1 Response options also include “unknown” and, where appropriate, “refused”.

**Table 2.** Substances that will be recorded if documented on toxicology testing during the hospitalization.

Substances of Interest		
Amphetamine	Cocaine metabolites	Opiates
Barbiturates	Ethanol	Oxycodone
Benzodiazepine	Fentanyl	Phencyclidine
Cannabinoids	Methadone	Other

**Table 3.** DSM-5 psychiatric comorbidities to be assessed via the NETSCID-5 interview.

Disorder	Disorder
Schizophrenia	Posttraumatic Stress Disorder
Schizoaffective Disorder	Acute Stress Disorder
Bipolar I Disorder	Insomnia Disorder
Bipolar II Disorder	Alcohol Use Disorder
Cyclothymic Disorder	Cannabis Use Disorder
Major Depressive Disorder	Phencyclidine Use Disorder
Persistent Depressive Disorder (dysthymia)	Inhalant Use Disorder
Panic Disorder	Opioid Use Disorder
Agoraphobia	Sedative, Hypnotic, Or Anxiolytic Use Disorder
Generalized Anxiety Disorder	Stimulant Use Disorder
	Tobacco Use Disorder

## ABBREVIATIONS

AICD: automatic implantable cardioverter defibrillator
BNP: brain natriuretic peptide
BUN: blood urea nitrogen
CABG: coronary artery bypass graft surgery
COPD: chronic obstructive pulmonary disease
DSM-5: Diagnostic and Statistical Manual, 5th edition
eGFR: estimated glomerular filtration rate
EMR: electronic medical record
ESC: European Society of Cardiology
HF: heart failure
HFmrEF: heart failure with mid-range ejection fraction
HFpEF: heart failure with preserved ejection fraction
HFrEF: heart failure with reduced ejection fraction
HIV/AIDS: human immunodeficiency virus/acquired immunodeficiency syndrome
LVAD: left ventricular assist device
LVEF: left ventricular ejection fraction
MI: myocardial infarction
MD: major depression
NHLBI: National Heart, Lung, and Blood Institute
NT-proBNP: N-terminal pro-brain natriuretic peptide
NYHA: New York Heart Association
OSA: obstructive sleep apnea
PCI: percutaneous coronary intervention
PSS: Perceived Stress Scale
PTSD: posttraumatic stress disorder
SBT: Short Blessed Test
SDOH: social determinants of health
TB: tuberculosis

## References

[1] Benjamin EJ, Muntner P, Alonso A, Bittencourt MS, Callaway CW, Carson AP, Heart Disease and Stroke Statistics-2019 Update: A Report From the American Heart Association. Circulation. 2019;139(10):e56–528. 30700139 10.1161/CIR.0000000000000659

[2] Ezekowitz JA. Management of comorbidities in heart failure. In: Mann DL, Felker GM, editors. Heart failure: A companion to Braunwald’s heart disease. 3rd ed. Philadelphia (PA, US): Elsevier-Saunders; 2016. p. 711–22.

[3] Forman DE, Rich MW, Alexander KP, Zieman S, Maurer MS, Najjar SS, Cardiac care for older adults. Time for a new paradigm. J Am Coll Cardiol. 2011;57(18):1801–10. 21527153 10.1016/j.jacc.2011.02.014 PMC4942282

[4] Rich MW, Chyun DA, Skolnick AH, Alexander KP, Forman DE, Kitzman DW, Knowledge Gaps in Cardiovascular Care of the Older Adult Population: A Scientific Statement From the American Heart Association, American College of Cardiology, and American Geriatrics Society. Circulation. 2016;133(21):2103–22. 27067230 10.1161/CIR.0000000000000380

[5] Eisele M, Adam W, Rakebrandt A, Boczor S, Blozik E, Trader JM, Importance of comorbidities in the treatment of primary care patients with heart failure-Baseline results of the observational RECODE-HF Study. Fam Pract. 2018;35(4):481–7. 29385434 10.1093/fampra/cmx135

[6] Tromp J, Tay WT, Ouwerkerk W, Teng TK, Yap J, MacDonald MR, Multimorbidity in patients with heart failure from 11 Asian regions: A prospective cohort study using the ASIAN-HF registry. PLoS Med. 2018;15(3):e1002541. 29584721 10.1371/journal.pmed.1002541 PMC5870945

[7] Lawson CA, Solis-Trapala I, Dahlstrom U, Mamas M, Jaarsma T, Kadam UT, Comorbidity health pathways in heart failure patients: A sequences-of-regressions analysis using cross-sectional data from 10,575 patients in the Swedish Heart Failure Registry. PLoS Med. 2018;15(3):e1002540. 29584734 10.1371/journal.pmed.1002540 PMC5870940

[8] Echouffo-Tcheugui JB, Xu H, DeVore AD, Schulte PJ, Butler J, Yancy CW, Temporal trends and factors associated with diabetes mellitus among patients hospitalized with heart failure: Findings from Get With The Guidelines-Heart Failure registry. Am Heart J. 2016;182:9–20. 27914505 10.1016/j.ahj.2016.07.025

[9] Freedland KE, Hesseler MJ, Carney RM, Steinmeyer BC, Skala JA, Davila-Roman VG, Major Depression and Long-Term Survival of Patients With Heart Failure. Psychosom Med. 2016;78(8):896–903. 27187847 10.1097/PSY.0000000000000346 PMC5067963

[10] Vart P, Matsushita K, Rawlings AM, Selvin E, Crews DC, Ndumele CE, SES, Heart Failure, and N-terminal Pro-b-type Natriuretic Peptide: The Atherosclerosis Risk in Communities Study. Am J Prev Med. 2018;54(2):229–36. 29241718 10.1016/j.amepre.2017.10.014 PMC5828682

[11] Dupre ME, Gu D, Xu H, Willis J, Curtis LH, Peterson ED. Racial and Ethnic Differences in Trajectories of Hospitalization in US Men and Women With Heart Failure. J Am Heart Assoc. 2017;6(11):e006290. 29146613 10.1161/JAHA.117.006290 PMC5721744

[12] Woldman S Multimorbidity in heart failure patients. Eur Heart J Qual Care Clin Outcomes. 2018;4(1):4–5. 29045585 10.1093/ehjqcco/qcx035

[13] Horton R Syndemics: health in context. Lancet. 2017;389(10072):881. 28271823 10.1016/S0140-6736(17)30640-2

[14] Boyd C, Smith CD, Masoudi FA, Blaum CS, Dodson JA, Green AR, Decision Making for Older Adults With Multiple Chronic Conditions: Executive Summary for the American Geriatrics Society Guiding Principles on the Care of Older Adults With Multimorbidity. J Am Geriatr Soc. 2019;67(4):665–73. 30663782 10.1111/jgs.15809

[15] Chen J, Normand SL, Wang Y, Krumholz HM. National and regional trends in heart failure hospitalization and mortality rates for Medicare beneficiaries, 1998-2008. JAMA. 2011;306(15):1669–78. 22009099 10.1001/jama.2011.1474 PMC3688069

[16] Chen J, Dharmarajan K, Wang Y, Krumholz HM. National trends in heart failure hospital stay rates, 2001 to 2009. J Am Coll Cardiol. 2013;61(10):1078–88. 23473413 10.1016/j.jacc.2012.11.057 PMC3939721

[17] Lafata JE, Pladevall M, Divine G, Ayoub M, Philbin EF. Are there race/ethnicity differences in outpatient congestive heart failure management, hospital use, and mortality among an insured population? Med Care. 2004;42(7):680–9. 15213493 10.1097/01.mlr.0000129903.12843.fc

[18] Sangaralingham LR, Shah ND, Yao X, Roger VL, Dunlay SM. Incidence and Early Outcomes of Heart Failure in Commercially Insured and Medicare Advantage Patients, 2006 to 2014. Circ Cardiovasc Qual Outcomes. 2016;9(3):332–7. 27166206 10.1161/CIRCOUTCOMES.116.002653 PMC4871725

[19] Rathore SS, Masoudi FA, Wang Y, Curtis JP, Foody JM, Havranek EP, Socioeconomic status, treatment, and outcomes among elderly patients hospitalized with heart failure: findings from the National Heart Failure Project. Am Heart J. 2006;152(2):371–8. 16875925 10.1016/j.ahj.2005.12.002 PMC2790269

[20] Foraker RE, Rose KM, Suchindran CM, Chang PP, McNeill AM, Rosamond WD. Socioeconomic status, Medicaid coverage, clinical comorbidity, and rehospitalization or death after an incident heart failure hospitalization: Atherosclerosis Risk in Communities cohort (1987 to 2004). Circ Heart Fail. 2011;4(3):308–16. 21430286 10.1161/CIRCHEARTFAILURE.110.959031 PMC3098576

[21] Verma AK, Schulte PJ, Bittner V, Keteyian SJ, Fleg JL, Pina IL, Socioeconomic and partner status in chronic heart failure: Relationship to exercise capacity, quality of life, and clinical outcomes. Am Heart J. 2017;183:54–61. 27979042 10.1016/j.ahj.2016.10.007

[22] Witte KK, Patel PA, Walker AMN, Schechter CB, Drozd M, Sengupta A, Socioeconomic deprivation and mode-specific outcomes in patients with chronic heart failure. Heart. 2018;104(12):993–8. 29386325 10.1136/heartjnl-2017-312539 PMC5992368

[23] Leventhal AM, Bello MS, Galstyan E, Higgins ST, Barrington-Trimis JL. Association of Cumulative Socioeconomic and Health-Related Disadvantage With Disparities in Smoking Prevalence in the United States, 2008 to 2017. JAMA Intern Med. 2019;179(6):777–85. 31009023 10.1001/jamainternmed.2019.0192 PMC6547249

[24] Allen LA, Smoyer Tomic KE, Smith DM, Wilson KL, Agodoa I. Rates and predictors of 30-day readmission among commercially insured and Medicaid-enrolled patients hospitalized with systolic heart failure. Circ Heart Fail. 2012;5(6):672–9. 23072736 10.1161/CIRCHEARTFAILURE.112.967356 PMC3873644

[25] Zingmond DS, Liang LJ, Parikh P, Escarce JJ. The Impact of the Hospital Readmissions Reduction Program across Insurance Types in California. Health Serv Res. 2018;53(6):4403–15. 29740816 10.1111/1475-6773.12869 PMC6232438

[26] Smolderen KG, Spertus JA, Tang F, Oetgen W, Borden WB, Ting HH, Treatment differences by health insurance among outpatients with coronary artery disease: insights from the national cardiovascular data registry. J Am Coll Cardiol. 2013;61(10):1069–75. 23375933 10.1016/j.jacc.2012.11.058 PMC3641586

[27] Rutledge T, Reis VA, Linke SE, Greenberg BH, Mills PJ. Depression in heart failure a meta-analytic review of prevalence, intervention effects, and associations with clinical outcomes. J Am Coll Cardiol. 2006;48(8):1527–37. 17045884 10.1016/j.jacc.2006.06.055

[28] Freedland KE, Rich MW, Skala JA, Carney RM, Davila-Roman VG, Jaffe AS. Prevalence of depression in hospitalized patients with congestive heart failure. Psychosom Med. 2003;65(1):119–28. 12554823 10.1097/01.psy.0000038938.67401.85

[29] Freedland KE, Carney RM, Rich MW, Steinmeyer BC, Skala JA, Davila-Roman VG. Depression and multiple rehospitalizations in patients with heart failure. Clin Cardiol. 2016;39(5):257–62. 26840627 10.1002/clc.22520 PMC4879082

[30] Jiang W, Alexander J, Christopher E, Kuchibhatla M, Gaulden LH, Cuffe MS, Relationship of depression to increased risk of mortality and rehospitalization in patients with congestive heart failure. Arch Intern Med. 2001;161(15):1849–56. 11493126 10.1001/archinte.161.15.1849

[31] Mentz RJ, Babyak MA, Bittner V, Fleg JL, Keteyian SJ, Swank AM, Prognostic significance of depression in blacks with heart failure: insights from Heart Failure: a Controlled Trial Investigating Outcomes of Exercise Training. Circ Heart Fail. 2015;8(3):497–503. 25901047 10.1161/CIRCHEARTFAILURE.114.001995 PMC4439310

[32] Adams J, Kuchibhatla M, Christopher EJ, Alexander JD, Clary GL, Cuffe MS, Association of depression and survival in patients with chronic heart failure over 12 Years. Psychosomatics. 2012;53(4):339–46. 22281436 10.1016/j.psym.2011.12.002 PMC3731067

[33] Deveney TK, Belnap BH, Mazumdar S, Roliman BL. The prognostic impact and optimal timing of the Patient Health Questionnaire depression screen on 4-year mortality among hospitalized patients with systolic heart failure. Gen Hosp Psychiatry. 2016;42:9–14. 27638965 10.1016/j.genhosppsych.2016.06.005 PMC5088502

[34] Fan H, Yu W, Zhang Q, Cao H, Li J, Wang J, Depression after heart failure and risk of cardiovascular and all-cause mortality: a meta-analysis. Prev Med. 2014;63:36–42. 24632228 10.1016/j.ypmed.2014.03.007

[35] Frasure-Smith N, Lesperance F, Habra M, Talajic M, Khairy P, Dorian P, Elevated depression symptoms predict long-term cardiovascular mortality in patients with atrial fibrillation and heart failure. Circulation. 2009;120(2):134–40, 3p following 40. 19564557 10.1161/CIRCULATIONAHA.109.851675

[36] Jiang W, Kuchibhatla M, Cuffe MS, Christopher EJ, Alexander JD, Clary GL, Prognostic value of anxiety and depression in patients with chronic heart failure. Circulation. 2004;110(22):3452–6. 15557372 10.1161/01.CIR.0000148138.25157.F9

[37] Milani RV, Lavie CJ, Mehra MR, Ventura HO. Impact of exercise training and depression on survival in heart failure due to coronary heart disease. Am J Cardiol. 2011;107(1):64–8. 21146688 10.1016/j.amjcard.2010.08.047

[38] Alosco ML, Spitznagel MB, Miller L, Raz N, Cohen R, Sweet LH, Depression is associated with reduced physical activity in persons with heart failure. Health Psychol. 2012;31(6):754–62. 22924448 10.1037/a0028711 PMC3610332

[39] Conley S, Feder S, Redeker NS. The relationship between pain, fatigue, depression and functional performance in stable heart failure. Heart Lung. 2015;44(2):107–12. 25576085 10.1016/j.hrtlng.2014.07.008 PMC4352387

[40] Friedman MM, Griffin JA. Relationship of physical symptoms and physical functioning to depression in patients with heart failure. Heart Lung. 2001;30(2):98–104. 11248712 10.1067/mhl.2001.114180

[41] Hawkins MA, Dolansky MA, Schaefer JT, Fulcher MJ, Gunstad J, Redle JD, Cognitive Function in Heart Failure Is Associated With Nonsomatic Symptoms of Depression But Not Somatic Symptoms. J Cardiovasc Nurs. 2015;30(5):E9–e17. 25055077 10.1097/JCN.0000000000000178 PMC4303562

[42] Gathright EC, Dolansky MA, Gunstad J, Redle JD, Josephson RA, Moore SM, The impact of medication nonadherence on the relationship between mortality risk and depression in heart failure. Health Psychol. 2017;36(9):839–47. 28726471 10.1037/hea0000529 PMC5573609

[43] Tang HY, Sayers SL, Weissinger G, Riegel B. The role of depression in medication adherence among heart failure patients. Clin Nurs Res. 2014;23(3):231–44. 23548500 10.1177/1054773813481801 PMC4130342

[44] Cameron J, Worrall-Carter L, Riegel B, Lo SK, Stewart S. Testing a model of patient characteristics, psychologic status, and cognitive function as predictors of self-care in persons with chronic heart failure. Heart Lung. 2009;38(5):410–8. 19755191 10.1016/j.hrtlng.2008.11.004

[45] Jonkman NH, Westland H, Groenwold RH, Agren S, Atienza F, Blue L, Do Self-Management Interventions Work in Patients With Heart Failure? An Individual Patient Data Meta-Analysis. Circulation. 2016;133(12):1189–98. 26873943 10.1161/CIRCULATIONAHA.115.018006 PMC5180429

[46] Garfield LD, Scherrer JF, Hauptman PJ, Freedland KE, Chrusciel T, Balasubramanian S, Association of anxiety disorders and depression with incident heart failure. Psychosom Med. 2014;76(2):128–36. 24434950 10.1097/PSY.0000000000000027 PMC3946309

[47] De Jong MJ, Chung ML, Wu JR, Riegel B, Rayens MK, Moser DK. Linkages between anxiety and outcomes in heart failure. Heart Lung. 2011;40(5):393–404. 21453974 10.1016/j.hrtlng.2011.02.002 PMC3149715

[48] Sokoreli I, de Vries JJ, Pauws SC, Steyerberg EW. Depression and anxiety as predictors of mortality among heart failure patients: systematic review and meta-analysis. Heart Fail Rev. 2016;21(1):49–63. 26572543 10.1007/s10741-015-9517-4

[49] Friedmann E, Thomas SA, Liu F, Morton PG, Chapa D, Gottlieb SS. Relationship of depression, anxiety, and social isolation to chronic heart failure outpatient mortality. Am Heart J. 2006;152(5):940.e1–8. 17070164 10.1016/j.ahj.2006.05.009

[50] Muller-Tasch T, Lowe B, Lossnitzer N, Frankenstein L, Tager T, Haass M, Anxiety and self-care behaviour in patients with chronic systolic heart failure: A multivariate model. Eur J Cardiovasc Nurs. 2018;17(2):170–7. 28718661 10.1177/1474515117722255

[51] Roy SS, Foraker RE, Girton RA, Mansfield AJ. Posttraumatic stress disorder and incident heart failure among a community-based sample of US veterans. Am J Public Health. 2015;105(4):757–63. 25713943 10.2105/AJPH.2014.302342 PMC4358172

[52] Taylor-Clift A, Holmgreen L, Hobfoll SE, Gerhart JI, Richardson D, Calvin JE, Traumatic stress and cardiopulmonary disease burden among low-income, urban heart failure patients. J Affect Disord. 2016;190:227–34. 26519644 10.1016/j.jad.2015.09.023 PMC4685032

[53] Sareen J, Cox BJ, Stein MB, Afifi TO, Fleet C, Asmundson GJ. Physical and mental comorbidity, disability, and suicidal behavior associated with posttraumatic stress disorder in a large community sample. Psychosom Med. 2007;69(3):242–8. 17401056 10.1097/PSY.0b013e31803146d8

[54] Spitzer C, Barnow S, Volzke H, John U, Freyberger HJ, Grabe HJ. Trauma, posttraumatic stress disorder, and physical illness: findings from the general population. Psychosom Med. 2009;71(9):1012–7. 19834051 10.1097/PSY.0b013e3181bc76b5

[55] Lu MLR, De Venecia TA, Goyal A, Rodriguez Ziccardi M, Kanjanahattakij N, Shah MK, Psychiatric conditions as predictors of rehospitalization among African American patients hospitalized with heart failure. Clin Cardiol. 2017;40(11):1020–5. 28750156 10.1002/clc.22760 PMC6490576

[56] Hendrie HC, Lindgren D, Hay DP, Lane KA, Gao S, Purnell C, Comorbidity profile and healthcare utilization in elderly patients with serious mental illnesses. Am J Geriatr Psychiatry. 2013;21(12):1267–76. 24206938 10.1016/j.jagp.2013.01.056 PMC3572246

[57] Ramsey CM, Leoutsakos JM, Mayer LS, Eaton WW, Lee HB. History of manic and hypomanic episodes and risk of incident cardiovascular disease: 11.5 year follow-up from the Baltimore Epidemiologic Catchment Area Study. J Affect Disord. 2010;125(1-3):35–41. 20570367 10.1016/j.jad.2009.12.024 PMC2922989

[58] Jorgensen M, Mainz J, Egstrup K, Johnsen SP. Quality of Care and Outcomes of Heart Failure Among Patients With Schizophrenia in Denmark. Am J Cardiol. 2017;120(6):980–5. 28774428 10.1016/j.amjcard.2017.06.027

[59] Ahmed AA, Patel K, Nyaku MA, Kheirbek RE, Bittner V, Fonarow GC, Risk of Heart Failure and Death After Prolonged Smoking Cessation: Role of Amount and Duration of Prior Smoking. Circ Heart Fail. 2015;8(4):694–701. 26038535 10.1161/CIRCHEARTFAILURE.114.001885 PMC5499230

[60] Del Gobbo LC, Kalantarian S, Imamura F, Lemaitre R, Siscovick DS, Psaty BM, Contribution of Major Lifestyle Risk Factors for Incident Heart Failure in Older Adults: The Cardiovascular Health Study. JACC Heart Fail. 2015;3(7):520–8. 26160366 10.1016/j.jchf.2015.02.009 PMC4508377

[61] Fonarow GC, Abraham WT, Albert NM, Stough WG, Gheorghiade M, Greenberg BH, A smoker’s paradox in patients hospitalized for heart failure: findings from OPTIMIZE-HF. Eur Heart J. 2008;29(16):1983–91. 18487210 10.1093/eurheartj/ehn210

[62] Shah AM, Pfeffer MA, Hartley LH, Moye LA, Gersh BJ, Rutherford JD, Risk of all-cause mortality, recurrent myocardial infarction, and heart failure hospitalization associated with smoking status following myocardial infarction with left ventricular dysfunction. Am J Cardiol. 2010;106(7):911–6. 20854949 10.1016/j.amjcard.2010.05.021

[63] Gah K, Spaderna H, Smits JM, Bramstedt KA, Weidner G. Smoking Status at Time of Listing for a Heart Transplant Predicts Mortality on the Waiting List: A Multicenter Prospective Observational Study. Prog Transplant. 2016;26(2):117–21. 27207399 10.1177/1526924816640687

[64] Snow SC, Fonarow GC, Ladapo JA, Washington DL, Hoggatt KJ, Ziaeian B. National Rate of Tobacco and Substance Use Disorders Among Hospitalized Heart Failure Patients. Am J Med. 2019 4;132(4):478–88.e4. 30562497 10.1016/j.amjmed.2018.11.038 PMC6615901

[65] Regan TJ. Alcohol and the cardiovascular system. JAMA. 1990;264(3):377–81. 2194048

[66] Walsh CR, Larson MG, Evans JC, Djousse L, Ellison RC, Vasan RS, Alcohol consumption and risk for congestive heart failure in the Framingham Heart Study. Ann Intern Med. 2002;136(3):181–91. 11827493 10.7326/0003-4819-136-3-200202050-00005

[67] Afonso L, Mohammad T, Thatai D. Crack whips the heart: a review of the cardiovascular toxicity of cocaine. Am J Cardiol. 2007;100(6):1040–3. 17826394 10.1016/j.amjcard.2007.04.049

[68] Awtry EH, Philippides GJ. Alcoholic and cocaine-associated cardiomyopathies. Prog Cardiovasc Dis. 2010;52(4):289–99. 20109599 10.1016/j.pcad.2009.11.004

[69] Diercks DB, Fonarow GC, Kirk JD, Jois-Bilowich P, Hollander JE, Weber JE, Illicit stimulant use in a United States heart failure population presenting to the emergency department (from the Acute Decompensated Heart Failure National Registry Emergency Module). Am J Cardiol. 2008;102(9):1216–9. 18940295 10.1016/j.amjcard.2008.06.045

[70] Neeki MM, Kulczycki M, Toy J, Dong F, Lee C, Borger R, Frequency of Methamphetamine Use as a Major Contributor Toward the Severity of Cardiomyopathy in Adults </=50 Years. Am J Cardiol. 2016;118(4):585–9. 27374605 10.1016/j.amjcard.2016.05.057

[71] Sliman S, Waalen J, Shaw D. Methamphetamine-Associated Congestive Heart Failure: Increasing Prevalence and Relationship of Clinical Outcomes to Continued Use or Abstinence. Cardiovascular toxicology. 2016;16(4):381–9. 26661075 10.1007/s12012-015-9350-y

[72] Nguyen P, Kamran H, Nasir S, Chan W, Shah T, Deswal A, Comparison of Frequency of Cardiovascular Events and Mortality in Patients With Heart Failure Using Versus Not Using Cocaine. Am J Cardiol. 2017;119(12):2030–4. 28456314 10.1016/j.amjcard.2017.03.034

[73] Gupta T, Mujib M, Agarwal P, Prakash P, Garg A, Sharma N, Association Between Opioid Abuse/Dependence and Outcomes in Hospitalized Heart Failure Patients. Am J Ther. 2016;23(2):e350–6. 25611362 10.1097/MJT.0000000000000190

[74] Braunstein JB, Anderson GF, Gerstenblith G, Weller W, Niefeld M, Herbert R, Noncardiac comorbidity increases preventable hospitalizations and mortality among Medicare beneficiaries with chronic heart failure. J Am Coll Cardiol. 2003;42(7):1226–33. 14522486 10.1016/s0735-1097(03)00947-1

[75] Qiu C, Winblad B, Marengoni A, Klarin I, Fastbom J, Fratiglioni L. Heart failure and risk of dementia and Alzheimer disease: a population-based cohort study. Arch Intern Med. 2006;166(9):1003–8. 16682574 10.1001/archinte.166.9.1003

[76] Rusanen M, Kivipelto M, Levalahti E, Laatikainen T, Tuomilehto J, Soininen H, Heart diseases and long-term risk of dementia and Alzheimer’s disease: a population-based CAIDE study. J Alzheimers Dis. 2014;42(1):183–91. 24825565 10.3233/JAD-132363

[77] Cermakova P, Lund LH, Fereshtehnejad SM, Johnell K, Winblad B, Dahlstrom U, Heart failure and dementia: survival in relation to types of heart failure and different dementia disorders. Eur J Heart Fail. 2015;17(6):612–9. 25581033 10.1002/ejhf.222 PMC4674979

[78] Adelborg K, Horvath-Puho E, Ording A, Pedersen L, Sorensen HT, Henderson VW. Heart failure and risk of dementia: a Danish nationwide population-based cohort study. Eur J Heart Fail. 2017;19(2):253–60. 27612177 10.1002/ejhf.631 PMC5522185

[79] Mirkin KA, Enomoto LM, Caputo GM, Hollenbeak CS. Risk factors for 30-day readmission in patients with congestive heart failure. Heart Lung. 2017;46(5):357–62. 28801110 10.1016/j.hrtlng.2017.06.005

[80] Mahajan SM, Burman P, Newton A, Heidenreich PA. A Validated Risk Model for 30-Day Readmission for Heart Failure. Stud Health Technol Inform. 2017;245:506–10. 29295146

[81] Davis JD, Olsen MA, Bommarito K, LaRue SJ, Saeed M, Rich MW, All-payer analysis of heart failure hospitalization 30-day readmission: comorbidities matter. Am J Med. 2017;130(1):93.e9–28. 10.1016/j.amjmed.2016.07.030 PMC5482409 27592085

[82] Manemann SM, Chamberlain AM, Boyd CM, Gerber Y, Dunlay SM, Weston SA, Multimorbidity in Heart Failure: Effect on Outcomes. J Am Geriatr Soc. 2016;64(7):1469–74. 27348135 10.1111/jgs.14206 PMC4943753

[83] Ather S, Chan W, Bozkurt B, Aguilar D, Ramasubbu K, Zachariah AA, Impact of noncardiac comorbidities on morbidity and mortality in a predominantly male population with heart failure and preserved versus reduced ejection fraction. J Am Coll Cardiol. 2012;59(11):998–1005. 22402071 10.1016/j.jacc.2011.11.040 PMC4687406

[84] Conrad N, Judge A, Tran J, Mohseni H, Hedgecott D, Crespillo AP, Temporal trends and patterns in heart failure incidence: a population-based study of 4 million individuals. Lancet. 2018;391(10120):572–80. 29174292 10.1016/S0140-6736(17)32520-5 PMC5814791

[85] Chamberlain AM, St Sauver JL, Gerber Y, Manemann SM, Boyd CM, Dunlay SM, Multimorbidity in heart failure: a community perspective. Am J Med. 2015;128(1):38–45. 25220613 10.1016/j.amjmed.2014.08.024 PMC4282820

[86] Christiansen MN, Kober L, Weeke P, Vasan RS, Jeppesen JL, Smith JG, Age-Specific Trends in Incidence, Mortality, and Comorbidities of Heart Failure in Denmark, 1995 to 2012. Circulation. 2017;135(13):1214–23. 28174193 10.1161/CIRCULATIONAHA.116.025941

[87] Centers for Medicare & Medicaid Services. Chronic Condition Charts. Baltimore (MD, US): Centers for Medicare & Medicaid Services; 2017.

[88] Singer M, Snipes C. Generations of suffering: experiences of a treatment program for substance abuse during pregnancy. Journal of Health Care for the Poor and Underserved. 1992;3(1):222–34. 1327239

[89] Singer M AIDS and the health crisis of the U.S. urban poor; the perspective of critical medical anthropology. Soc Sci Med. 1994;39(7):931–48. 7992126 10.1016/0277-9536(94)90205-4

[90] Centers for Disease Control. CDC Grand Rounds: the TB/HIV syndemic. MMWR Morb Mortal Wkly Rep. 2012;61(26):484–9. 22763886

[91] Singer M, Bulled N, Ostrach B, Mendenhall E. Syndemics and the biosocial conception of health. Lancet. 2017;389(10072):941–50. 28271845 10.1016/S0140-6736(17)30003-X

[92] Ponikowski P, Voors AA, Anker SD, Bueno H, Cleland JGF, Coats AJS, 2016 ESC Guidelines for the diagnosis and treatment of acute and chronic heart failure: The Task Force for the diagnosis and treatment of acute and chronic heart failure of the European Society of Cardiology (ESC)Developed with the special contribution of the Heart Failure Association (HFA) of the ESC. Eur Heart J. 2016;37(27):2129–200. 27206819 10.1093/eurheartj/ehw128

[93] Katzman R, Brown T, Fuld P, Peck A, Schechter R, Schimmel H. Validation of a short Orientation-Memory-Concentration Test of cognitive impairment. Am J Psychiatry. 1983;140(6):734–9. 6846631 10.1176/ajp.140.6.734

[94] Rockwood K, Song X, MacKnight C, Bergman H, Hogan DB, McDowell I, A global clinical measure of fitness and frailty in elderly people. CMAJ. 2005; 173(5) :489–95. 16129869 10.1503/cmaj.050051 PMC1188185

[95] Hammond G, Johnston K, Huang K, Joynt Maddox KE. Social determinants of health improve predictive accuracy of clinical risk models for cardiovascular hospitalization, annual cost, and death. Circ Cardiovasc Qual Outcomes. 2020;13(6):e006752. 32412300 10.1161/CIRCOUTCOMES.120.006752 PMC7299736

[96] Palacio A, Mansi R, Seo D, Suarez M, Garay S, Medina H, Social determinants of health score: does it help identify those at higher cardiovascular risk? Am J Manag Care. 2020;26(10):e312–8. 33094943 10.37765/ajmc.2020.88504

[97] Rhee TG, Marottoh RA, Cooney LM Jr, Fortinsky RH. Associations of Social and Behavioral Determinants of Health Index with self-rated health, functional limitations, and health services use in older adults. J Am Geriatr Soc. 2020;68(8):1731–8. 32227645 10.1111/jgs.16429

[98] Cohen S, Kamarck T, Mermelstein R. A global measure of perceived stress. J Health Soc Behav. 1983;24(4):385–96. 6668417

[99] Brodey BB, First M, Finthicum J, Haman K, Sasiela JW, Ayer D. Validation of the NetSCID: an automated web-based adaptive version of the SCID. Compr Psychiatry. 2016;66:67–70. 26995238 10.1016/j.comppsych.2015.10.005 PMC4800162

[100] American Psychiatric Association. DSM-5 Task Force. Diagnostic and statistical manual of mental disorders: DSM-5. 5th ed. Washington (DC, US): American Psychiatric Association; 2013.

[101] Riegel B, Barbaranelh C, Carlson B, Sethares KA, Daus M, Moser DK, Psychometric Testing of the Revised Self-Care of Heart Failure Index. J Cardiovasc Nurs. 2019;34(2):183–92. 30303894 10.1097/JCN.0000000000000543 PMC7179813

[102] Hays RD, Bjorner JB, Revicki DA, Spritzer KF, Celia D. Development of physical and mental health summary scores from the patient-reported outcomes measurement information system (PROMIS) global items. Qual Life Res. 2009;18(7):873–80. 19543809 10.1007/s11136-009-9496-9 PMC2724630

[103] Gruber-Baldini AL, Velozo C, Romero S, Shulman LM. Validation of the PROMIS((R)) measures of self-efficacy for managing chronic conditions. Qual Life Res. 2017;26(7):1915–24. 28239781 10.1007/s11136-017-1527-3 PMC5479750

[104] Harris PA, Taylor R, Thielke R, Payne J, Gonzalez N, Conde JG. Research electronic data capture (REDCap)—a metadata-driven methodology and workflow process for providing translational research informatics support. J Biomed Inform. 2009;42(2):377–81. 18929686 10.1016/j.jbi.2008.08.010 PMC2700030

[105] Liu D, Schaubel DE, Kalbfleisch JD. Computationally efficient marginal models for clustered recurrent event data. Biometrics. 2012;68(2):637–47. 21957989 10.1111/j.1541-0420.2011.01676.x PMC3384760

[106] Carlson B, Pozehl B, Hertzog M, Zimmerman L, Riegel B. Predictors of overall perceived health in patients with heart failure. J Cardiovasc Nurs. 2013;28(3):206–15. 22495800 10.1097/JCN.0b013e31824987a8 PMC3396769

